# Cultural differences in vocal expression analysis: Effects of task, language, and stimulus-related factors

**DOI:** 10.1371/journal.pone.0275915

**Published:** 2022-10-10

**Authors:** Shuyi Zhang, Marc D. Pell

**Affiliations:** School of Communication Sciences and Disorders, McGill University, Montréal, Canada; Polytechnic Institute of Coimbra: Instituto Politecnico de Coimbra, PORTUGAL

## Abstract

Cultural context shapes the way that emotions are expressed and socially interpreted. Building on previous research looking at cultural differences in judgements of facial expressions, we examined how listeners recognize speech-embedded emotional expressions and make inferences about a speaker’s feelings in relation to their *vocal* display. Canadian and Chinese participants categorized vocal expressions of emotions (anger, fear, happiness, sadness) expressed at different intensity levels in three languages (English, Mandarin, Hindi). In two additional tasks, participants rated the intensity of each emotional expression and the intensity of the speaker’s feelings from the same stimuli. Each group was more accurate at recognizing emotions produced in their native language (in-group advantage). However, Canadian and Chinese participants both judged the speaker’s feelings to be equivalent or more intense than their actual display (especially for highly aroused, negative emotions), suggesting that similar inference rules were applied to vocal expressions by the two cultures in this task. Our results provide new insights on how people categorize and interpret speech-embedded vocal expressions versus facial expressions and what cultural factors are at play.

## Introduction

Though basic emotion states from the face and voice can be recognized cross-culturally [1–4], social variables regulate how and when people communicate their emotions nonverbally and what types of inferences they draw from emotion expressions [5–7]. To investigate how culture influences emotional judgement, and to build on previous work focusing on facial expressions [8], this study compared how Canadian and Chinese adults evaluate *vocally* expressed emotions in spoken language and whether they make similar inferences about the speaker’s inner feelings based on their emotional display.

### Culture and emotion expression

Display rules refer to the norms that guide socially appropriate behaviors in everyday communication [9]. According to cross-cultural research on language and emotion, Western independent cultures emphasize personal feelings, uniqueness, and autonomy; within these societies, people tend to prefer a direct and expressive style of communication [10, 11]. On the other hand, the core values of Eastern Asian interdependent cultures are social relations and cooperation [7, 10, 12]. To promote social interdependence, East Asians are more likely to communicate indirectly and suppress or down-regulate their emotional expression, especially for socially disruptive emotions, to preserve harmony and avoid conflicts [8, 13, 14].

A study of Olympic athletes appears to exemplify this effect: compared to athletes from independent cultures, who preferred more direct expressions, those from interdependent cultures tended to mask their facial expression of emotions to a greater extent during the prize presentation [15]. Social norms also influence the frequency and intensity of smiles across cultures [16]. Compared to American leaders who expressed more intense smiles, Chinese leaders preferred to smile more calmly [16]. The influence of cultural rules on specific regulation tendencies can even be found in infants [17]. According to Camras and colleagues [17], Chinese infants produced less frequent smiles and cries compared to European American and Japanese participants, who were more reactive and expressive. Interestingly, despite sharing an interdependent and hierarchical orientation, Japanese infants behaved significantly different from Chinese infants in this study, with greater expressivity overall. This evidence suggests that emotional communication practices (e.g., the intensity and content of emotional expression) are likely to vary even for groups that share similar processes for cultural construal.

Thus, cultural rules dictating the extent to which senders control their emotional displays may lead to differences in how *intensely* members of independent vs. interdependent cultures (also known as individualistic vs. collectivistic cultures) typically express their emotions in daily interactions. In turn, this could affect how expressions of emotion are evaluated by observers to infer the subjective experience of another’s emotion [8]. In a key study, Matsumoto et al. [8] compared how American and Japanese adults infer the intensity of another person’s emotional feeling based on their facial expression. Participants categorized Japanese and Caucasian faces [8] conveying high and low intensity expressions of four emotions (anger, happiness, sadness, surprise) and then rated the intensity of the facial expression and the sender’s internal feelings. For high intensity displays of emotion, American participants rated external (facial) expressions as more intense than internal feelings, suggesting that they assumed the sender was actually feeling less inside. For low intensity emotions, Japanese participants were unique in that they rated the internal feelings of senders as more intense than their expression, indicating that they assumed senders were restraining their expression of emotions (cultural differences in other conditions such as high or very high intensity were nonsignificant, suggesting that cultural effects were modulated in distinct ways by the emotional intensity of facial expressions). These patterns were taken as evidence that cultural values significantly impact the way that people attribute affective states to others based on the form of their emotional expression [8].

While these and other studies [18–21] exemplify that cultural knowledge modulates emotion perception for *faces*, research on the *voice* is far less advanced. Human speech contains both the linguistic message and acoustically rich vocal signals that people utilize to exchange social information and to share emotions [22]. As people produce an utterance, acoustic features (e.g., changes in pitch, loudness, and duration of speech elements) dynamically reveal the speaker’s emotional state over time [6, 23], promoting pancultural recognition of most basic emotions in the voice [1–3, 24]. According to Scherer et al. [1], participants from Europe, America, and Asia could all identify vocally expressed emotions in German (anger, sadness, fear, joy) with an accuracy of 66%, much greater than expected by chance (see Pell et al. [2] for similar data). In addition, vocal expressions of negative emotions (especially sadness and anger) tend to have higher recognition accuracy than that of joy/happiness [2, 25, 26]. It should be noted that many researchers have presented linguistically anomalous ‘pseudo-utterances’ rather than natural speech in voice studies. This is because pseudo-utterances are speech-embedded expressions that can be effectively controlled to express emotions in a culturally appropriate manner without providing meaningful linguistic cues to the listener, isolating any effects due to vocal attributes in speech [1, 2, 26].

Evidence that the vocal emotion communication is shaped by culture-specific behaviors and communicative rules—what Elfenbein and Ambady [27] refer to as “cultural dialects”—is now beginning to accrue [6, 24, 28]. Using forced-choice tasks, many studies have reported an in-group advantage for recognizing vocal emotions expressed in a participant’s native versus foreign language (see Laukka & Elfenbein [29] for a recent meta-analysis). Like the literature on facial expression, these patterns suggest that the ability to apply inference rules to identify the emotional meaning of vocal displays is more accurate when listeners are familiar with culturally dictated modes of vocal expression in the native language. However, it is not clear whether the ability to make graded affective judgements about vocal displays, and to use this information to infer how the speaker is *feeling*, would vary according to the individual’s cultural orientation (independent vs. interdependent) and related behavioral conventions. Thus, expanding the work of Matsumoto and colleagues [8] to determine how observers evaluate a speaker’s internal feelings (vs. external display) based on their *vocal* expression would represent an important step forward in this literature.

### Objectives

To advance knowledge of cross-cultural emotion perception, here we chose two disparate cultural groups, Chinese and Canadian, who are often taken as typical examples of interdependent and independent cultures [30, 31]. We then compared how adults from these two groups judge speech-embedded emotions and use vocal information to infer the intensity of a speaker’s feelings. Adapting the design of Matsumoto and colleagues [8], individuals from Canada and Mainland China rated vocal expressions of emotion produced with different levels of intensity, expressed in their native and two non-native languages. Participants rated both the intensity of the vocal expression and the intensity of the speaker’s feelings in different language and emotion conditions. They also performed a “classic” forced-choice emotion recognition task across languages to benchmark our results to existing research using this paradigm.

Based on the literature, we predicted that Canadian and Chinese participants would each demonstrate above chance recognition of vocally expressed emotions across languages, but that each group would display greater sensitivity (i.e., accuracy) for emotions expressed in their native language (English for Canadians, Mandarin for Chinese, “in-group advantage”). Of greater theoretical interest, given that East Asian cultures tend to emphasize emotion control compared to North American cultures, in which the emotional authenticity and expression of personal feelings are more valued [8], we hypothesized that Chinese participants would rate vocal emotion expressions as less intense than they rated the speaker’s internal feelings, an effect that could be more pronounced when emotional intensity in the voice is low. In contrast, Canadians may infer that speakers are feeling less intense emotions than they vocally express in speech, especially when vocal intensity is high, as they tend to express emotions more visibly in daily life [8].

## Methods

### Participants

We estimated the required sample size with a priori power analysis [32]. To achieve a statistical power of 0.8 and an expected effect size of 0.3 (Cohen’s *d*), a minimum of 42 participants were needed [8]. In our study, 49 Canadian (M_age_ = 22.08 years, SD = 3.73) and Chinese (M_age_ = 23.43 years, SD = 2.73) participants between 18 and 35 years of age were recruited through online advertisement. Two participants who failed to adhere to task instructions were subsequently excluded, leaving 47 participants (24 Canadian, 23 Chinese) for statistical analysis. All participants were recruited and tested in Montreal, Canada. Canadians spoke English as their native language, whereas Chinese participants grew up in mainland China and spoke Mandarin as their mother tongue (most Chinese participants also spoke English with various levels of proficiency). Chinese participants were international students or immigrants who had recently moved to Montreal. To minimize effects of emotional acculturation (e.g., recalibration of emotional displays) due to exposure to Western cultures [33, 34], none of the Chinese participants had been outside of China for more than two years (M = 1.11 years, Range = 0.5–2 years). The study has been ethically approved by McGill Faculty of Medicine Institutional Review Board. Informed written consents were obtained from all individual participants prior to the start of the experiment.

### Materials

Stimuli were short pseudo-utterances expressing emotions produced by native speakers of English, Mandarin, and Hindi, selected from perceptually validated recording databases [26, 35]. Pseudo-utterances were approximately 1–2 seconds in duration and contained both pseudo content words and function words which made the sentence meaningless, although the target emotion was expressed clearly (e.g., 他们在楼谷中投玩 (Mandarin), I nestered the flugs (English), 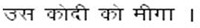 (Hindi)). Given that the study was conducted in an English-speaking country and Chinese participants knew English, another language that was completely foreign to both groups (Hindi) was included as a third condition.

For each language (English, Mandarin, Hindi), we selected pseudo-utterances produced by two male and two female speakers which expressed four different emotions (anger, fear, happiness, sadness), each communicated with high and low intensity. Full details of stimulus recording and validation procedures are supplied elsewhere [26, 35]. In brief, the emotion encoders were native speakers of each language who had lay experience in broadcasting, acting or vocal performance. All sound stimuli were recorded digitally in a sound-attenuating chamber, and encoders were instructed to produce utterances as naturally as possible to reliably convey the intended emotion. After the collection and editing of sound files, utterances were validated by a group of native listeners (24 English speakers, 24 Mandarin speakers, and 20 Hindi speakers) who were required to categorize the emotional meaning (7-alternative forced-choice task) and rate the intensity of each emotional stimulus (this process was conducted separately for each language). The resulting perceptual data reflecting the clarity and intensity of emotional expressions as judged by native listeners of each language were used to select appropriate stimuli for the current experiment [26, 35].

For each language and emotion, stimuli judged with the highest recognition rates by native listeners were selected, while controlling as much as possible for the sex of the speaker and linguistic features of the pseudo-utterance within and across language conditions (mean recognition rates across emotions and languages ranged from 53% to 98%, where chance performance was 14%). Due to how each language inventory was originally constructed, native emotion recognition was somewhat lower in the Hindi condition overall (see Table 1). To ensure a good separation between high and low intensity expressions of each emotion, we calculated the average intensity rating for all stimuli in each language/emotion condition and then categorized items as high or low intensity based on whether the individual rating was above (or below) the native group average. Overall, 288 sound stimuli were used: 3 languages (English, Mandarin, Hindi) x 4 emotions (anger, fear, happiness, sadness) x 2 intensity levels (high, low) x 12 items (3 linguistically distinct pseudo-utterances x 4 speakers).

**Table 1:** Perceptual features of high and low intensity vocal expressions selected for the experiment.

Emotion	Intensity	Emotional Target Recognition (Proportions correct)			Emotional Intensity Rating (1 = not at all, 5 = very much)		
		Mandarin	English	Hindi	Mandarin	English	Hindi
Anger	High	0.89	0.94	0.69	3.78	3.84	3.42
	Low	0.84	0.89	0.64	3.00	2.91	2.56
Fear	High	0.91	0.95	0.64	3.78	3.95	3.24
	Low	0.80	0.91	0.53	2.86	3.06	2.59
Happiness	High	0.84	0.86	0.78	3.38	3.29	3.52
	Low	0.80	0.76	0.68	2.43	2.25	2.51
Sadness	High	0.87	0.98	0.84	3.76	3.89	3.66
	Low	0.86	0.93	0.78	2.92	3.17	2.77
**Total**		**0.85**	**0.90**	**0.70**	**3.24**	**3.30**	**3.04**

Data show the mean recognition accuracy and perceived intensity of emotional expression judged by native listeners of each language based on earlier validation studies [26, 35].*

*Mandarin stimuli were perceptually evaluated by a group of 24 native listeners [35]. English and Hindi stimuli were judged by a group of 24 and 20 native listeners, respectively [26]. Methods for constructing and perceptually rating stimuli in these two studies were highly comparable (see source articles for details).

### Experimental procedures and measurements

Participants were tested in a quiet research laboratory in a group of maximum four people. Upon arrival, questionnaires were administered to obtain basic demographic information (e.g., age, education, and language proficiency). Participants were seated comfortably in front of a computer screen and heard auditory stimuli over high-quality headphones controlled by SuperLab 6.0 presentation software (Cedrus, U.S.), which also collected participant responses. Both oral and written instructions about the experimental procedures were provided in the participant’s *native language* (English for the Canadian group, Mandarin for the Chinese group). For the first Recognition task, participants listened to each utterance and categorized the expression as one of five possible emotions (anger, fear, happiness, sadness, neutral) based on the emotion they heard (Instruction: What emotion did you hear?). Once they clicked the appropriate response option on the computer screen, a new screen appeared and participant rated the intensity level of the speaker’s vocal expressions on a scale of 1 to 8 (1 = not at all and 8 = very much; Instruction for the Display task: What is the intensity level of the speaker’s vocal expression?).

After the completion of the Recognition and Display tasks, participants performed a Feeling task in which they listened to the same utterances in a different order and rated the intensity of what they thought the speaker was feeling by clicking on the corresponding number on a scale of 1–8 (Instruction: What is the intensity level that you think the speaker is actually feeling?). Participants were advised that the utterances they would hear would “not make sense” and that they should make their judgements based on the speaker’s voice. Each auditory stimulus was played twice in the experiment, and stimuli were organized into blocks of 36 trials separated by a mandatory break (blocks were randomized for presentation order). The basic structure of each trial was: a fixation cross (500 milliseconds/ms), the auditory stimulus, a visual scale which presented response options on the computer screen (until a response was executed), and a 1500ms inter-trial interval. The display task was always presented before the Feeling task in order to keep the methodology comparable with Matsumoto’s [8] study with facial expressions.

To familiarize participants with different stimulus types, voices, and response procedures, they first performed six practice trials composed of two stimuli from each language (English, Mandarin, and Hindi) expressing different emotions (anger, fear, happiness, and sadness) and intensities (high and low). During the practice phase, instructions could be clarified, and any volume or sound quality issues could be adjusted before starting the experiment. The whole experiment lasted approximately 1.5 hours and participants were paid CAD $15 for their participation.

### Statistical analysis

Our study is a within-subjects design (with repeated-measures) in which all participants complete the same tasks. To better account for variances and multiple sources of random effects, a series of linear mixed effects models (LMM), instead of ANOVA employed in Matsumoto et al.’s study [8], were used in our study. Analysis of the Recognition task considered the accuracy of emotional target recognition in each language condition after calculating unbiased *Hu* scores from the raw hit rates [36]. The model included Culture (Canadian, Chinese), Emotion (Anger, Fear, Happiness, Sadness), Language (English, Mandarin, Hindi), and Intensity (High, Low) as fixed factors, participant sex (male, female) as a control factor, and subject as a random factor to account for individual differences. For the Display and Feeling tasks, the mean intensity rating (scale = 1–8) served as the dependent variable. In these models, the evaluative Task (Display, Feeling) was added as a fixed factor and Item of speech stimuli was included as an additional random factor. The lme4 package of R (version 1.2.5033) was used for running the analysis and the threshold for significance was set for 0.05.

## Results

### Recognition task

Table 2 shows emotional target recognition accuracy (*Hu* score) for participants in each group in the three language conditions. Overall, it can be seen that the accuracy of Canadian (0.57) and Chinese (0.61) participants was similar across the three language conditions, and that emotional expressions in Mandarin (0.66) and English (0.70) were recognized more accurately than in Hindi (0.42). Vocal expressions of sadness (0.69) had the highest recognition rates across languages, followed by anger (0.62), fear (0.54), and happiness (0.51). In general, participants displayed higher accuracy when judging high (0.65) versus low (0.53) intensity vocal expressions.

**Table 2:** Emotional target recognition accuracy (*Hu* scores) for Canadian and Chinese participants when listening to vocal emotion expressions in Mandarin, English and Hindi.

Vocal Expression		Participant Culture/Stimulus Language					
Emotion	Intensity	English Canadian			Mandarin Chinese		
		Mandarin	English	Hindi	Mandarin	English	Hindi
Anger	High	0.61	0.88	0.44	0.83	0.77	0.47
	Low	0.64	0.73	0.34	0.73	0.63	0.37
Fear	High	0.57	0.82	0.32	0.78	0.64	0.36
	Low	0.52	0.75	0.23	0.74	0.54	0.23
Happiness	High	0.42	0.85	0.46	0.87	0.63	0.53
	Low	0.23	0.56	0.27	0.69	0.29	0.32
Sadness	High	0.67	0.83	0.61	0.84	0.79	0.63
	Low	0.61	0.74	0.50	0.79	0.70	0.58
**Total**		**0.53**	**0.77**	**0.40**	**0.78**	**0.62**	**0.44**

Shaded cells refer to the ‘in-group’ condition for each participant group.

**Fig 1:**
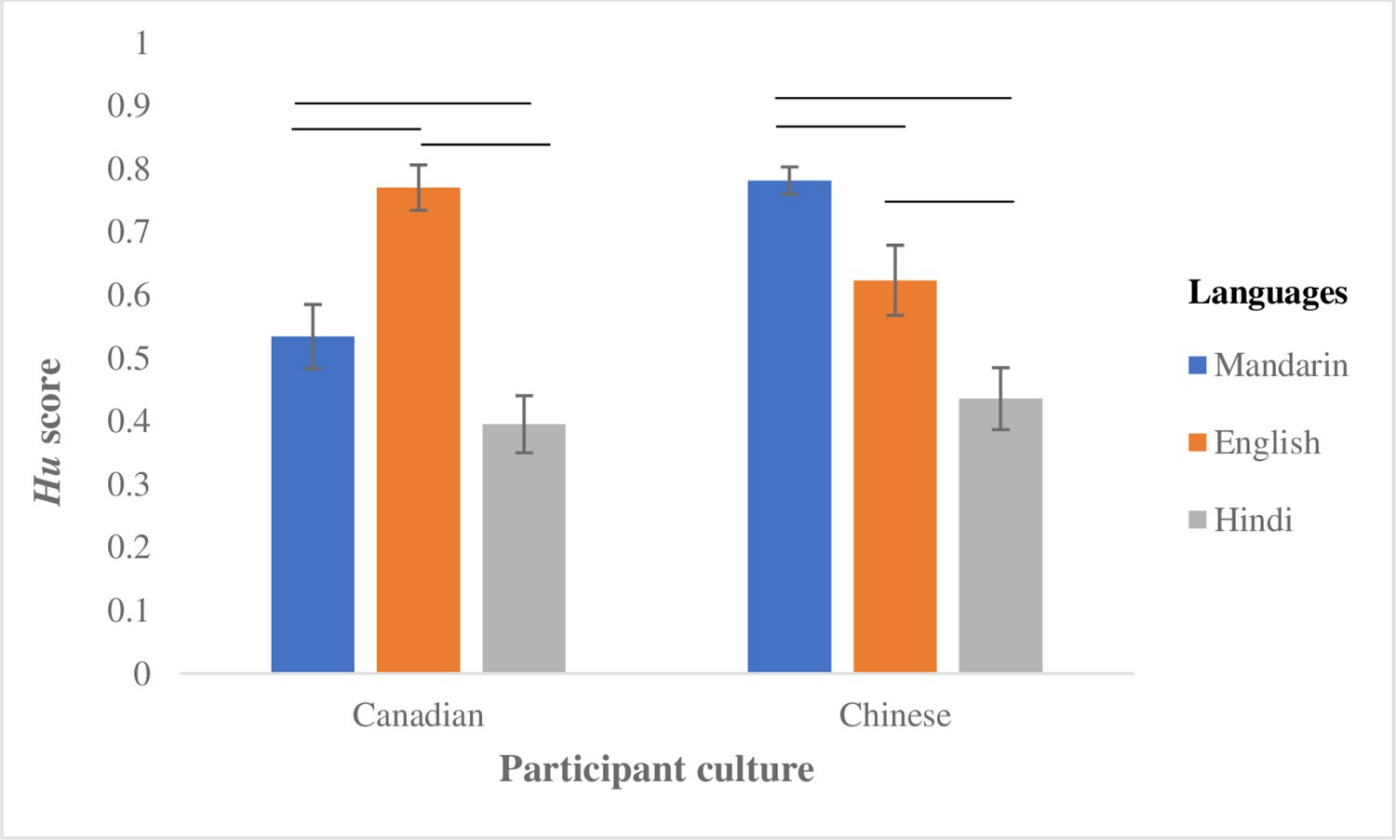
Effects of participant culture and language on vocal emotion recognition. The black lines above the bar graphs represent significant comparisons between different languages.

The LMM on *Hu* scores indicated that emotion recognition varied significantly by Emotion, F (3,1032) = 85.04, *p* < 0.001, Language, F (2,1032) = 400.43, *p* < 0.001, and Stimulus Intensity, F(1,1032) = 180.50, *p* < 0.001, but there was no main effect of Culture on accuracy performance, F (1,1032) = 2.46, *p* = 0.12, *ns*. Rather, performance of the two cultural groups was informed by interactions among the stimulus variables, as highlighted below. First, as hypothesized there was an in-group advantage in vocal emotion recognition for each cultural group (Culture x Language, F (2,1032) = 166.83, *p* < 0.001). Post-hoc analysis showed that vocal expressions in Mandarin (*b* = 0.34, *SE* = 0.02, *t* = 17.58, *p* < 0.001) and English (*b* = 0.19, *SE* = 0.02, *t* = 9.55, *p* < 0.001) were identified with significantly higher accuracy overall than in Hindi. Canadian participants displayed better recognition of emotions expressed in English than in Mandarin (*b* = 0.24, *SE* = 0.02, *t* = 12.06, *p* < 0.001) and Hindi (*b* = 0.38, *SE* = 0.02, *t* = 19.16, *p* < 0.001), and also performed better in Mandarin vs. Hindi (*b* = 0.14, *SE* = 0.02, *t* = 7.10, *p* < 0.001). By contrast, Chinese participants displayed better recognition of emotions in Mandarin than English (*b* = 0.16, *SE* = 0.02, *t* = 8.18, *p* < 0.001) and Hindi (*b* = 0.35, *SE* = 0.02, *t* = 17.91, *p* < 0.001), and also performed better in English than Hindi (*b* = 0.19, *SE* = 0.02, *t* = 9.72, *p* < 0.001). An illustration of the in-group effect on recognition accuracy is presented in Fig 1.

Cultural patterns of recognition further depended on the emotional meaning of the vocal expression (Culture x Emotion, F (3,1032) = 3.96, *p* = 0.008, Culture x Emotion x Language, F (6,1032) = 12.80, *p* < 0.001). In broad terms, results showed that happy vocal expressions were recognized less reliably than all other emotions, with slight differences across languages and between cultural groups (see Table 2). Across languages, Canadian participants judged happy expressions less accurately than anger (*b* = -0.14, *SE* = 0.03, *t* = -5.04, *p* < 0.001), fear (*b* = -0.07, *SE* = 0.03, *t* = -2.54, *p* = 0.01) and sad expressions (*b* = -0.20, *SE* = 0.03, *t* = -6.98, *p* < 0.001). Chinese participants identified happy expressions less accurately than angry (*b* = -0.08, *SE* = 0.03, *t* = -2.90, *p* = 0.004) and sad expressions (*b* = -0.17, *SE* = 0.03, *t* = -6.18, *p* < 0.001), but happy did not differ from fear (*p =* 0.79). When emotion-specific patterns were examined between groups in each language condition, they only varied when judging expressions in Mandarin. When listening to Mandarin, Canadian participants recognized happiness less reliably than anger (*b* = -0.30, *SE* = 0.03, *t* = -9.51, *p* < 0.001), fear (*b* = -0.22, *SE* = 0.03, *t* = -7.08, *p* < 0.001), and sadness (*b* = -0.31, *SE* = 0.03, *t* = -10, *p* < 0.001), whereas there were no significant differences among emotions for Chinese participants (*p’s >* 0.1). Interestingly, while there was an overall effect of stimulus intensity on emotion recognition accuracy (high > low), the categorical intensity of the stimulus did not interact with Culture (*F* = 1.02, *p* = 0.31) nor with any of the stimulus variables (*F’s <* 0.72, *p’s >* 0.63).

### Display vs. feeling ratings

The mean group intensity ratings of vocal expressions in the two evaluative tasks (Display, Feeling) are provided in Table 3, by language, emotion, and stimulus intensity category. Focusing first on effects of our experimental manipulations on perceived intensity ratings, the LMM revealed main effects of Language, F (2,26610) = 93.31, *p* < 0.001, stimulus Intensity, F (1, 26610) = 54.65, *p* < 0.001, and Emotion, F (3,26610) = 43.00, *p* < 0.001 across the two tasks. Intensity ratings were generally higher when evaluating expressions in Mandarin (Canadian: *b* = 0.91, *SE* = 0.10, *t* = 9.31, *p* < 0.001; Chinese: *b* = 1.03, *SE* = 0.11, *t* = 9.27, *p* < 0.001) and English (Canadian: *b* = 0.73, *SE* = 0.10, *t* = 7.52, *p* < 0.001; Chinese: *b* = 0.90, *SE* = 0.11, *t* = 8.14, *p* < 0.001) than in Hindi (overall ratings for Mandarin and English did not differ, *p* = 0.07). Predictably, participants gave higher ratings to high vs. low intensity vocal expressions (Canadian group: *b* = 0.34, *SE* = 0.09, *t* = 3.89, *p* < 0.001; Chinese group: *b* = 0.47, *SE* = 0.10, *t* = 4.58, *p* < 0.001). Also, anger and fear expressions were perceived as more intense than happy/sad expressions overall (*p’s* < 0.001).

**Table 3:** Average intensity ratings when judging the vocal expression (display task) and the speaker’s internal experience (feeling task), for Canadian and Chinese participants.

Stimulus	Stimulus	English Canadian			Mandarin Chinese		
Emotion	Intensity	Mandarin	English	Hindi	Mandarin	English	Hindi
**Display task (vocal expression)**							
Anger	High	5.92	6.18	4.78	5.88	6.23	4.68
	Low	5.55	5.28	4.57	5.35	5.37	4.18
Fear	High	5.03	5.84	4.47	5.52	5.70	4.45
	Low	4.56	5.06	4.22	4.78	4.96	4.11
Happiness	High	4.93	5.18	4.66	5.93	4.66	4.41
	Low	4.67	4.67	4.30	5.00	4.30	4.12
Sadness	High	4.88	4.75	4.23	5.53	5.14	4.11
	Low	4.62	4.31	4.13	5.05	4.47	3.75
Total		**5.02**	**5.16**	**4.42**	**5.38**	**5.10**	**4.23**
**Feeling task (internal experience)**							
Anger	High	6.34	6.35	5.09	6.15	6.25	4.97
	Low	5.94	5.75	4.67	5.67	5.70	4.47
Fear	High	5.54	5.94	4.79	5.91	6.19	4.75
	Low	5.27	5.53	4.50	5.29	5.66	4.37
Happiness	High	5.30	4.77	4.63	5.47	4.72	4.50
	Low	5.05	4.46	4.37	4.71	4.08	4.27
Sadness	High	5.15	4.75	4.23	5.32	5.16	4.14
	Low	4.86	4.49	3.90	5.03	4.80	3.86
Total		**5.43**	**5.26**	**4.52**	**5.44**	**5.32**	**4.42**

**Fig 2:**
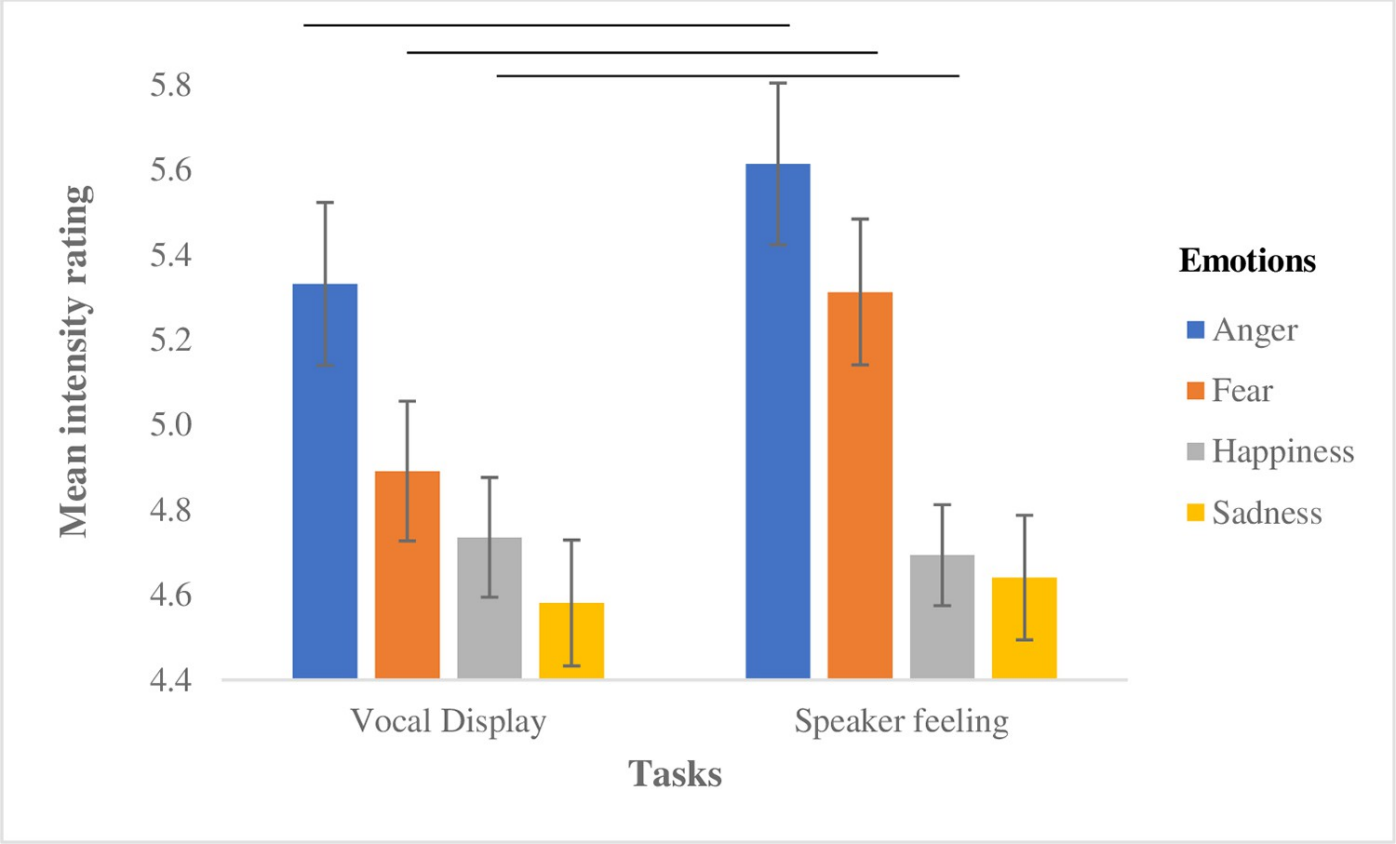
Perceived intensity of vocal expressions by emotion type for the display and feeling tasks. The black lines above the bar graphs represent significant comparisons between different emotions.

Of key interest, ratings varied significantly according to the evaluative Task, F (1,26610) = 129.33, *p* < 0.001, and were generally higher when judging a speaker’s feeling than their vocal expression. This effect differed depended on the emotion expression under scrutiny, Task x Emotion: F (3,26610) = 43.09, *p* < 0.001. Participants assigned significantly higher ratings to speaker feelings than to their vocal expression when listening to anger (*b* = -0.26, *SE* = 0.04, *t* = -5.94, *p* < 0.001) and fear (*b* = -0.44, *SE* = 0.04, *t* = -10.45, *p* < 0.001). Happiness, on the other hand, displayed the inverse relationship (display > feeling, *b* = 0.11, *SE* = 0.04, *t* = 2.48, *p* = 0.01). The evaluative task did not influence ratings for sadness (*p* = 0.35) (Fig 2).

Turning to the effects of culture on the intensity ratings, there was no main effect of Culture on intensity ratings overall (*p* = 0.95), but there was a significant four-way interaction of Culture x Task x Language x Emotion, F (6, 26610) = 4.63, *p* < 0.001. The intensity ratings of vocal displays differed across tasks and the emotional meanings of expressions. For vocal expression ratings (display task), both groups gave significant higher ratings to expressions in anger than all other emotions, including fear (*b* = -0.37, *SE* = 0.17, *t* = -2.16, *p* = 0.03), happiness (*b* = -1.05, *SE* = 0.17, *t* = -6.16, *p* <0.001), and sadness (*b* = -1.09, *SE* = 0.17, *t* = -6.40, *p* = <0.001). They also assumed that speakers felt more intense anger than happiness (*b* = -1.50, *SE* = 0.16, *t* = -9.35, *p* <0.001) and sadness (*b* = -1.22, *SE* = 0.16, *t* = -7.56, *p* <0.001) in the feeling task; however, the intensity ratings of anger and fear did not differ significantly (*b* = -0.19, *SE* = 0.16, *t* = -1.15, *p* = 0.25).

Independent of the evaluative task, there were cultural differences in intensity ratings due to changes in stimulus-related variables: Culture x Language x Emotion, F (6,26610) = 12.67, *p* < 0.001; Culture x Language x Emotion x Intensity, F (6, 26610) = 2.46, *p* = 0.02. Inspection of the 4-way interaction showed that Canadian participants gave higher ratings to expressions in Mandarin and English than Hindi for all emotions (all *p’s* < 0.04), whereas Chinese participants rated expressions in Mandarin and English as more intense than Hindi only for angry, fearful, and sad voices (all *p’s* < 0.01). Interestingly, Chinese participants assigned higher intensity ratings to happy expressions produced in Mandarin vs. English (*b* = -0.84, *SE* = 0.18, *t* = -4.57, *p* < 0.001), whereas no significant differences were found for this contrast when judged by Canadians (*p* = 0.11).

## Discussion

People evaluate emotional information to make inferences about what others feel from both facial and vocal expressions, a process that is modulated by cultural knowledge and social rules [20, 37]. According to Matsumoto and colleagues [8], cultural differences between Eastern and Western cultures significantly influence how individual perceivers interpret facial expressions to reflect the sender’s emotional experience [38]. Using a similar approach but focusing on *vocal* expressions, here we discovered that Canadian and Chinese participants both rated the internal feelings of the speaker to be similar or stronger than their vocal displays (depending on the emotion), a pattern that was not dictated by the underlying intensity of the stimulus. Despite many similarities in how our two cultural groups rated vocal expressions under different tasks, Canadian and Chinese listeners each displayed a robust in-group advantage to *categorize* emotions more accurately in their native language, supporting previous research [2, 25, 29]. We elaborate on the potential significance of these observations below.

### Judging emotional intensity of vocal displays and speaker feelings

In the rating tasks (display vs. feelings), the perceived intensity of vocal expressions varied predictably according to emotion type; expressions of anger and fear were judged to be more intense than happiness and sadness, a pattern that was qualitatively similar in all three languages [2, 26, 35]. The language of vocal stimuli (English, Mandarin, and Hindi) also influenced the perceived intensity of emotions, with generally lower ratings assigned to vocal expressions in Hindi than in Mandarin/English by all participants. According to a previous study on voice emotion recognition [26], pseudo-utterances expressed in Hindi had a lower emotion recognition rate than English when judged by native decoders. These patterns likely reflect the signal clarity of emotional contrasts in the original production inventories, affecting how stimuli were perceived and selected (see Table 1 and Pell et al. [26]).

Of greater theoretical interest, when participants focused their attention in specific tasks (display vs. feelings), they tended to rate a speaker’s feelings to be more intense than their vocal expression, although this effect was largely driven by more intense emotions (“fear” and “anger”). Interestingly, both Canadian and Chinese participants seemed to infer that speakers were experiencing more intense feelings of anger and fear than conveyed by their vocal expression in speech. Thus, contrary to the results of Matsumoto et al. [8] for facial expressions, there was limited evidence that our cultural groups arrived at different conclusions about what a speaker was feeling based on their overt expression of emotion in the voice. Rather, the data suggest that all participants displayed similar sensitivity to emotional and linguistic dimensions of speech when evaluating the intensity of vocal expressions: they considered the intensity of a speaker’s feelings to be relatively equivalent or sometimes greater than the intensity of their actual vocal display, with the possible exception of happiness (which showed the opposite effect).

Our failure to replicate Matsumoto et al.’s [8] study when vocal expressions are presented first raises the question of how cultural moderating effects differ as a function of the expressive channel (face vs. voice) or sensory modality (visual vs. auditory) under study. Though both vocal and facial expressions contain rich information about one’s emotions and social intentions [39], each channel is associated with unique physiological attributes which impact on perception and social evaluation processes [25, 40, 41]. Some researchers argue that facial expressions contain more salient (less probabilistic) information about emotions than speech-embedded vocal expressions [42, 43], which could promote more nuanced inferences about experienced emotion when evaluating a face. Other work suggests that the channel of expression (i.e., face vs. voice) governs *which* emotions are perceived with greater clarity [44, 45], which could impact results of the two studies. Unlike static facial expressions [8], speech-embedded vocal displays unfold over time in conjunction with meaningful *linguistic* information [46]. Given the high demands of controlling the acoustic signal to maintain speech intelligibility, it is possible that listeners become habituated to encountering speech-embedded emotional expressions that are socially regulated or attenuated to preserve linguistic communication, especially for expressions linked to high arousal (fear, anger). Indeed, in situations in which speakers are “overcome” with emotion, they are typically unable to speak and produce non-linguistic vocal expressions (e.g., screams, laughter) instead of speech-embedded vocal expressions [45]. Acquired differences in how emotional displays of varying intensity are typically communicated in the face and in speech contexts could partly explain why our results do not align with those of Matsumoto et al. [8].

A second methodological difference of our study is that we recruited Canadian and Chinese participants as exemplars of ‘independent’ versus ‘interdependent’ cultures, whereas Matsumoto et al. [8] studied individuals from the United States and Japan. Similar to the Japanese culture, Chinese society endorses low independent cultural ethos and put more emphasize on emotion control [7, 47]. American and Canadian cultures are thought to be similar in that both groups have a relatively independent cultural ethos; however, based on recent work, it seems possible that Canadians and Americans also differ in their expressive style and how they evaluate social cues. In a study by Giles and colleagues [30], Canadian, Chinese, and German participants evaluated the politeness and appropriateness of prosocial lies as they watched videotaped conversations. It was expected that members from interdependent cultures (e.g., Chinese) would prefer implicit communication, such as prosocial lies, to preserve face [31, 48, 49], whereas members from independent cultures would favor direct communicative strategies (‘blunt truths’). However, the researchers found that both Canadian and Chinese participants preferred indirect communication and face-preservation strategies when compared to German participants, who favored blunt truths during the social interactions [30, 50]. This finding emphasizes that patterns of communication style and socioemotional processing vary along the dimensions of independent vs. interdependent, even for groups which purportedly share the same cultural orientation.

Although independent and interdependent orientations value different display rules (e.g., direct vs indirect expressions) [7, 51], the impact of those social orientations are also highly dependent on individuals’ self-construal [52]. It has been proposed that all people have an independent and interdependent self-construal, although one form of construal may dominate in specific contexts [52, 53]. When evaluating vocal expressions, our study supplies evidence that both Canadian and Chinese participants assumed that speakers express strong negative emotions (e.g., fear and anger) in a more suppressed manner while speaking. Possibly, this result implies that both groups endorse a more interdependent form of self-construal and prefer to use more indirect communicative strategies to avoid imposing on others or hurting their feelings [52]. In the future, more self-conscious emotions (e.g., pride, guilt) could be included to better determine how emotional evaluations are informed by individual and group construal processes [54].

The finding that participants expected speakers to attenuate their expressions of anger and fear when speaking may provide insight into display rules governing these and other emotions in the context of spoken language (although not in a culture-specific manner). According to research, the expression of positive emotions may enhance relationships, while the display of negative emotions could harm self-image and social reputation [55, 56]. Since emotion regulation, especially expression control for negative emotions, is extremely important for maintaining good social interactions and relationships, it is not uncommon for people to presume that others are masking/attenuating their display of angry emotions in order to maintain good self-perception and group harmony, regardless of their cultural backgrounds or the language they speak. The higher intensity ratings for speaker’s internal feelings of fear might also relate to the perceptions of social power and dominance [57]. During social interactions, people may not only decode emotions of others, but also make heuristic inferences about their interpersonal traits. Since the expression of fear is more likely to be perceived as a sign of submission and low social dominance [58, 59], people may intentionally suppress their expression of fear, which may have produced the pattern of results we observed.

Lastly, given that participants thought that vocal expressions of happiness and sadness more closely reflected their true subjective experience, it seems that neither Canadian nor Chinese adults assumed that speakers would socially modulate (e.g., mask/restrain) expressions of these emotions while speaking. This finding implies that the clarity of these vocal expressions in conveying one’s innermost feelings is relatively high for both cultural groups. According to a recent study by Salvador and colleagues [60], Latin Americans (an interdependent group) showed an emotionally expressive pattern, especially for positive/socially engaging emotions, to build social relationships and maintain group harmony. This evidence suggests that cultural norms may facilitate the expression of certain kinds of emotions, such as positive emotions to preserve harmony. Thus, based on the evidence above, the ‘unrestrained’ expression of vocal emotions might well be practical tools for Chinese and Canadian participants to promote harmonious communication and interdependence. These initial conclusions merit examination as this literature moves forward.

### Emotion recognition and the in-group advantage

Our report demonstrates that vocal emotion expressions are categorized at high accuracy levels, even in unfamiliar languages, supporting arguments that speech-embedded emotions possess invariant acoustic properties that are recognized pan culturally [2, 4, 29, 61]. Using a forced-choice paradigm, our cultural groups performed at a similar accuracy level overall (~60% correct target recognition across emotion/language conditions) and all emotions were recognized at levels exceeding chance (20%). Recognition of emotional utterances increased as stimulus intensity increased [8, 62], suggesting that discrete (posed) vocal emotions are more clearly differentiated when they are expressed at high intensity levels [40]. As expected, vocal expressions of negative emotions achieved systematically higher recognition levels for both cultural groups despite there being only one positive emotion under study [4, 22, 26]. Interestingly, while happiness was generally associated with low recognition across languages and stimulus types, this difference was less evident for the Chinese participants when judging stimuli in their native language, Mandarin. This finding could reflect the fact that positive, socially-engaging emotions are shaped to a greater extent by sociocultural learning [2, 63], and by extension, that individuals from interdependent (e.g., Chinese) cultures become more attuned to cultural expressions of socially engaging emotions that promote group bonding and interaction [56, 64].

Findings concurrently reveal a marked in-group accuracy advantage when Chinese and Canadian participants judged emotions in their *native* language (Mandarin for Chinese, English for Canadians, review Fig 1). Thus, while individuals from each cultural background exhibited many qualitatively similar tendencies in how they judged vocal expressions, listening to non-native vocal expressions ultimately hampered their ability to correctly identify the speaker’s emotion state. We interpret this result to reflect each group’s familiarity with fine-grained, culturally defined vocal features and characteristic of their own nonverbal dialect, which selectively facilitates emotion recognition in the native listening condition [6, 25, 29]. It should be noted that an in-group recognition advantage may also be observed for specific expressions of emotion [19].

The possibility that emotion recognition improves by knowing a *second* language—for example, by becoming familiar with cultural dialects for communicating emotion in the course of learning a second language—has been raised by some research [6, 61, 65]. While not our main purpose, our design allows commentary on this issue. For the Chinese participants who have some experience with English culture, accuracy was much better in English than in Hindi, suggesting that recognition performance might have improved due to their English second language proficiency [61]. According to meta-analyses of cross-cultural communication [25, 29], people’s frequency of social interactions with other groups influence the size of the in-group accuracy advantage. Moreover, the in-group recognition advantage decreases in individuals who have greater exposure to other cultures [2, 4, 24, 29]. Our data provides further evidence that the exposure to another culture may facilitate the perception and evaluation of emotional communication.

Similar to Chinese participants, Canadian participants also showed a clear accuracy advantage for Mandarin over Hindi, despite the fact that both non-native languages were entirely unfamiliar to most of them, and Mandarin is linguistically more distant from English than Hindi [66]. These patterns imply that recognition accuracy in non-native languages was not linked to linguistic familiarity or typology; rather, they were governed by the perceptual clarity of emotional contrasts in the original recording materials, and the extent to which non-native expressions possessed salient modal features associated with discrete emotions in the voice (which was generally lower for Hindi, Pell et al. [26]. We speculate that the strength and clarity of prototypical emotion displays at the production stage was largely responsible for how Chinese and Canadian participants performed in the non-native language conditions, where they could not draw upon knowledge of dialectal tendencies or conventions in emotion expression acquired in the native language [5, 25]. Thus, the idea that second language proficiency gives listeners an advantage in recognizing vocal emotions in that language remains a topic for further study.

### Future directions

In summary, our study supplies evidence that vocally expressed emotions possess invariant properties that allow them to be recognized cross-culturally, while highlighting that sociocultural “shaping” of emotions in speech leads to a robust advantage when listening to in-group members who share the same nonverbal dialect [6, 25, 29]. This recognition advantage persisted despite evidence that Chinese and Canadian participants displayed comparable sensitivity to affective dimensions (valence, arousal) and discrete emotional qualities of vocal expressions produced by multiple speakers in three language contexts. Of even greater novelty, our findings underscore that for vocal expressions, inferences derived about the intensity of a speaker’s feelings may have a relatively direct relationship to the intensity of their outward display, at least when spoken utterances are not presented in a richer social context; only when anger and fear were expressed did listeners assume that speakers were attenuating or ‘masking’ their actual feelings through their vocal expression [67]. While replication is clearly needed, our work on the voice contrasts with data reported by Matsumoto and colleagues [8], who found that people from East Asian and North American cultures interpret the significance of faces in culturally governed ways depending on the intensity of the expression.

When evaluating the intensity of vocal expressions and speaker feelings, our study is limited in terms of the naturalism of our speech materials. In real life social interactions, people tend to use both spontaneous and posed expressions to convey thoughts and emotions [68, 69]. Compared to naturally occurring spontaneous speech, simulated portrayals of vocal expressions may at times sound more intense or over-acted [62] which would affect interpretation of our results. However, participants in both groups rated vocal displays as similar or less intense than speaker feelings, implying that the acted expressions in our study may functions highly similar to those derived from natural speech. Future studies that record stimuli from spontaneous conversations would provide more insightful explanations to this finding. It would also be beneficial if researchers could include complex and socially (dis)engaging emotions (e.g., guilt and anger) to examine the relationship between culture, language, and emotion, and to further explore how inferences are drawn from emotional displays.

Another limitation of this study is related to potential cultural exposure effects on our Chinese participants. According to a culture and communication study [34], Chinese immigrants to Canada have shown similar behavioral responses to multisensory emotion processing with North American participants. This demonstrates that people can learn and adapt to new ways of emotional communication with sufficient experience with out-groups. Moreover, research have shown that East Asians can adopt more than one cultural identity [70–72], and that they are adept at switching between cultural frames depending on the social context or communicator (intra-individual cultural dynamics) [70, 73]. Since our study involved bilingual Chinese participants, we attempted to control the testing environment by providing instructions only in Chinese, given by a Chinese examiner (whereas Canadian participants completed the experiment in English). We also restricted the duration of time that Chinese (and Canadian) participants had spent outside their own cultural milieu, although this was still greater for the Chinese who were tested in Canada (similar to Matsumoto et al. [8] who tested Japanese participants in the USA). Ideally, future experiments would be performed in the home country of each cultural group to better control for potential effects of cultural exposure, immersive experiences, and the selection of participants who, owing to their choice to study or work abroad, could be more adept at fluctuating between cultural frames [74].

Using emotion judgement tasks that involved participants from Western and Eastern cultures, which are highly comparable with Matsumoto’s [8] study, our research demonstrates that cultures do not equally endorse interdependent/independent frames. Rather, the dimension of interdependent and independent may be relative and future studies are encouraged to take other social factors such as regions and religions into account. Instead of limiting interpretations to people from particular geopolitical regions, cultures should be viewed as more dynamic systems that are continuously evolving [73, 75, 76]. The rapid changes of cultural values and the interactions between them could have significant impacts on emotional communication and interpretative processes, as new practices and assumptions are incorporated into existing knowledge systems.
